# Studying the Endothelial Glycocalyx *in vitro*: What Is Missing?

**DOI:** 10.3389/fcvm.2021.647086

**Published:** 2021-04-14

**Authors:** Andrew B. Haymet, Nicole Bartnikowski, Emily S. Wood, Michael P. Vallely, Angela McBride, Sophie Yacoub, Scott B. Biering, Eva Harris, Jacky Y. Suen, John F. Fraser

**Affiliations:** ^1^Critical Care Research Group, The Prince Charles Hospital, Chermside, QLD, Australia; ^2^Faculty of Medicine, University of Queensland, St Lucia, QLD, Australia; ^3^Science and Engineering Faculty, Queensland University of Technology, Brisbane, QLD, Australia; ^4^Division of Cardiac Surgery, The Ohio State University Wexner Medical Center, Columbus, OH, United States; ^5^Department of Global Health and Infection, Brighton and Sussex Medical School, Brighton, United Kingdom; ^6^Oxford University Clinical Research Unit, Wellcome Trust Africa Asia Programme, Ho Chi Minh City, Vietnam; ^7^Centre for Tropical Medicine and Global Health, University of Oxford, Oxford, United Kingdom; ^8^Division of Infectious Diseases and Vaccinology, School of Public Health, University of California, Berkeley, Berkeley, CA, United States

**Keywords:** endothelium, glycocalyx, vasculopathy, vascular, endothelial surface layer

## Abstract

All human cells are coated by a surface layer of proteoglycans, glycosaminoglycans (GAGs) and plasma proteins, called the glycocalyx. The glycocalyx transmits shear stress to the cytoskeleton of endothelial cells, maintains a selective permeability barrier, and modulates adhesion of blood leukocytes and platelets. Major components of the glycocalyx, including syndecans, heparan sulfate, and hyaluronan, are shed from the endothelial surface layer during conditions including ischaemia and hypoxia, sepsis, atherosclerosis, diabetes, renal disease, and some viral infections. Studying mechanisms of glycocalyx damage *in vivo* can be challenging due to the complexity of immuno-inflammatory responses which are inextricably involved. Previously, both static as well as perfused *in vitro* models have studied the glycocalyx, and have reported either imaging data, assessment of barrier function, or interactions of blood components with the endothelial monolayer. To date, no model has simultaneously incorporated all these features at once, however such a model would arguably enhance the study of vasculopathic processes. This review compiles a series of current *in vitro* models described in the literature that have targeted the glycocalyx layer, their limitations, and potential opportunities for further developments in this field.

## Introduction

All cells in humans are coated by a surface layer of glycans called the glycocalyx (1, 2). This coating is a matrix consisting of various proteoglycans, glycosaminoglycans (GAGs), and plasma proteins, and it provides endothelial cellular mechano-sensation and transduction (3). Its principal GAGs include heparan sulfate (HS) and hyaluronic acid (HA), and core proteins primarily include syndecans and glypicans (1). The composition of the glycocalyx is in a state of constant flux, as it continuously replenishes components that are removed by flowing plasma (4). The glycocalyx offers a scaffold to which plasma proteins and GAGs may bind (4, 5), and remains an inactive structure until plasma constituents are bound, at which point it becomes the physiologically active endothelial surface layer (6).

In health, the glycocalyx performs a number of physiological functions, including the exertion of profound influence of shear stress on the vascular wall, maintenance of a selective permeability barrier and a low hydraulic conductivity, and modulation of adhesion of blood leukocytes and platelets (7). These responses are vital for the regulation of blood pressure, maintenance of tissue perfusion, and control of leukocyte recruitment during innate immune responses and inflammation (8). In particular, its constituent GAGs play a key role in cell-cell signaling and tissue injury. Innate immune cells, such as fibroblasts, mast cells, dendritic cells, monocytes and neutrophils recognize pathogenic invasion using pattern recognition receptors. When activated, these receptors drive signaling cascades which ultimately recruit leucocytes to the site of tissue injury (9). Furthermore, cytokines such as tumor necrosis factor (TNF) and interleukin 1 (IL1) promote leukocyte extravasation by increasing the levels of leukocyte adhesion molecules on endothelial cells (9). In a murine model by Wang et al. reducing sulphation by 60% with an endothelium-specific knockout of N-acetyl glucosamine N-deacetylase-N-sulfotransferase-1, which is required for the addition of sulfate to heparan sulfate chains, decreased neutrophil infiltration (10). This emphasizes the extent to which glycocalyx integrity modulates feedback between innate immune responses and inflammation.

The glycocalyx is also critically involved in angiogenesis. A study by Park-Windol et al. reported that *in vitro* knockout of endomucin-1, an integral membrane glycocalyx glycoprotein, reduced migration, inhibited cell growth without compromising cell survival, and suppressed tube morphogenesis of endothelial cells, whereas over-expression of endomucin-1 led to increased migration, proliferation and tube formation (11).

During homeostatic conditions, the glycocalyx lies in dynamic equilibrium between shedding of its components following the shear stress of blood flow, and *de novo* synthesis of its components, such as hyaluronic acid, by endothelial cells (12). The glycocalyx, being a dynamic structure, is also readily susceptible to injury. Destruction of the endothelial glycocalyx, which ranges from 200 to 2,000 nm in thickness, decreases vascular barrier function and leads to protein extravasation and tissue oedema, loss of substrate supply to tissues, and an increase in platelet and leucocyte adhesion (13). Major components of the glycocalyx, including syndecans, heparan sulfate, and hyaluronan, are shed from the endothelial surface layer under various acute and chronic clinical conditions, with the best characterized examples including ischaemia and hypoxia, sepsis, atherosclerosis, diabetes, renal disease, and haemorrhagic viral infections (7). Critically, heparan sulfate and syndecan-1 are released into the circulation during periods of ischaemia-reperfusion injury, and these levels reduce accordingly during therapeutic intervention (14). These may therefore be used as surrogate biomarkers of endothelial injury (14). Furthermore, syndecans have been shown to act as soluble messengers. Jannaway et al. demonstrated that thrombin can cleave syndecan 3/4 ectodomain into fragments which interact with endothelial cells, causing paracellular hyperpermeability (15). Through contact independent signaling, this vascular leakage may be amplified by perivascular cells, such as in the case of Dengue viral protein NS1 (16).

There has been a significant increase in academic interest in the role of the glycocalyx in cardiovascular pathophysiology. Previously, both static as well as perfused *in vitro* models have reported imaging data of glycocalyx components under flow, assessment of barrier function, or the interplay between blood components (e.g., leucocytes) and the glycocalyx. No model to date has combined all of these parameters, however this would be highly advantageous in the study of a vast array of pathophysiological states (e.g., infective vasculopathies, ischaemia-reperfusion injury, shock, sepsis, responses to foreign antigens). This paper aims to review current *in vitro* models which study glycocalyx pathophysiology, with an aim to identify areas of potential further development in this field.

## Studying the Endothelial Glycocalyx: Models and Imaging Techniques

Given the greatly increased level of interest in the endothelial glycocalyx in recent years, a wide range of *in vitro* models to study the glycocalyx layer have been developed, integrating a variety of imaging modalities. These models have been used to study numerous pathophysiological states, including hyperglycaemia, endotheliopathy of shock, and ischaemic preconditioning (17–19). A summary of selected *in vitro* models in the literature is given in Table 1.

**Table 1:** Summary of *in vitro* models that directly study glycocalyx pathophysiology.

**References**	**Model type**	**EC cell type**	**Flow condition**	**Model fluid**	**Research question/model details**	**Method of assessment of endothelial surface layer**	**Relevant outcomes**
***In vitro*** **(Transwell)**							
Diebel et al. (18)	*In vitro;* Transwell plates	HUVECs	Static	Cell media	Cells exposed to hydrogen peroxide and/or adrenaline to stimulate post-trauma/haemorrhagic shock. The effect of early administration of tranexamic acid (TXA) on mitigating endothelial damage was evaluated	***Permeability***: Transendothelial filtration of FITC-Dextran, intracellular adhesion molecule expression (ICAM). ***Biomarkers***: Syndecan-1 (glycocalyx degradation); soluble thrombomodulin (endothelial injury); tissue type plasminogen activator (tPA, profibrinolytic), plasminogen activator inhibitor-1 (PAI-1, antifibrinolytic); and angiopoietin-2/angiopoietin-1 ratio (APO-2/APO-1, vascular reactivity).	Antifibrinolytic and other protective effects of TXA administration on endothelial injury were time-dependent, supporting the concept that clinical efficacy of tranexamic acid requires “early administration.”
Martin et al. (20)	*In vitro;* Transwell plates	HUVECs	Static	Cell media	HUVECs were treated with varying concentrations of noraderenaline and adrenaline, and exposed to simulated shock conditions	***Imaging***: N/A. ***Biomarkers***: Syndecan-1 (glycocalyx degradation); thrombomodulin and angiopoietin-1/angiopoietin-2 ratios (endothelial integrity); tissue type plasminogen activator (tPA, profibrinolytic), and plasminogen activator inhibitor-1 (PAI-1, antifibrinolytic)	Noradrenaline induced a fibrinolytic state with elevated tPA levels compared to adrenaline. Both degraded the glycocalyx, however adrenaline appeared to produce more severe endothelial instability as demonstrated by increased APO-2 levels
Puerta-Guardo et al. (21)	*In vitro;* Transwell plates	Human pulmonary, dermal, and umbilical vein microvascular endothelial cells	Static	Cell media	Confluent human pulmonary microvascular endothelial cell (HPMECs) monolayers grown on gelatin-coated coverslips were exposed to different concentrations of Dengue virus (DENV) non-structural protein 1 (NS1)	***Immunofluorescence staining***: WGA lectin conjugated to Alexa 647 to stain N-acetyl neuraminic acid (Sia); anti-human heparanase 1, anti-human cathepsin L; anti-heparan sulfate proteoglycan 2 for perlecan, anti-human CD138 for syndecan-1; Neu1 antibody (H300): sc-32936; Neu2 antibody PA5-35114; Ganglioside sialidase antibody (N-18): sc-55826 for Neu3. ***Imaging***: Inverted fluorescence microscopy. ***Permeability***: Trans-endothelial electrical resistance, using an epithelial volt ohm meter with “chopstick” electrodes.	DENV NS1 disrupted the endothelial glycocalyx on human pulmonary microvascular endothelial cells, inducing degradation of sialic acid and shedding of heparan sulfate proteoglycans. NS1 also activated cathepsin L, a lysosomal cysteine proteinase, in endothelial cells, which activated heparanase via enzymatic cleavage
Puerta-Guardo et al. (22)	*In vitro/in vivo;* Transwell permeable supports/tissue culture plates	Human pulmonary, dermal, umbilical vein, brain, and liver endothelial cells	Static	Cell media	NS1 proteins added to confluent monolayers of human endothelial cells, and amount of NS1 bound to the surface of the endothelial cell monolayers was determined by immunofluorescence microscopy. Alterations in permeability assessed using TEER and solute flux assays	***Staining*****:** For sialic acid, wheat germ agglutinin (WGA) conjugated to Alexa Fluor 647 (5 μg/ml); for heparan sulfate, anti-heparan sulfate antibody (1:50 in 1X PBS, primary) and anti-mouse IgM Alexa Fluor 488 (1:100 in 1X PBS, secondary) ***Imaging:*** Inverted fluorescence microscopy ***Permeability:*** TEER of endothelial monolayers using Epithelial Volt Ohm Meter, and a solute flux assay using measured fluorescence of include fluorescein isothiocyanate (FITC).	NS1 proteins from dengue, Zika, West Nile, Japanese encephalitis, and yellow fever viruses selectively alter the permeability of monolayers of endothelial cell lines derived from distinct human tissues. Flavivirus NS1 proteins modulate endothelial permeability in a tissue-specific manner both *in vitro* and *in vivo*, potentially influencing flavivirus dissemination, pathogenesis, and disease
Wang et al. (23)	*In vitro/in vivo;* Transwell plates	HUVECs	Static	Cell media	Investigation of effect of platelet microparticles (PMPs) in diabetes on aortic vascular endothelial injury. *In vitro**:*** cells treated with platelet microparticles	***Imaging***: Wheat germ agglutinin (WGA) staining with laser confocal microscopy. ***Permeability***: Transendothelial filtration of FITC-conjugated dextran (70 kDa).	Increased PMPs levels contributed to aortic vascular endothelial injuries in diabetes through activating the mTORC1 pathway
Butler et al. (24)	*In vitro/in vivo;* tissue culture dishes	Human glomerular endothelial cells	10 dyn/cm^2^ shear stress using orbital shaker	Cell media, with added mannitol or NaCl (to avoid osmotic stress)	Investigation of the effect of salt and aldosterone exposure on pathologic remodeling of the glomerular glycocalyx. *In vitro:* cells exposed to 0.1 nM aldosterone and 145 mMol NaCl. *In vivo:* murine model of administration of 0.6 μg/g/d of aldosterone (subcutaneous minipump) and 1% NaCl drinking water	***Imaging***: Intravital multiphoton imaging. ***Immunofluorescence***: Primary antibodies using anti-heparan sulfate and anti-syndecan-4, followed by secondary antibodies	Reduction in cell surface glycocalyx components (heparan sulfate and syndecan-4) and disrupted shear sensing was observed consistent with damage of the glycocalyx, when cells were exposed to 0.1 nM aldosterone and 145 mMol NaCl. Targeting matrix metalloproteinases 2 and 9 with a specific gelatinase inhibitor preserved the glycocalyx
Glasner et al. (25)	*In vivo/in vitro;* Transwell permeable supports	Human dermal endothelial cells; human pulmonary microvascular endothelial cells	Static	Cell media	*In vivo* and *in vitro* examination of the relative contributions of inflammatory mediators and endothelial cell-intrinsic pathways in endothelial dysfunction induced by Dengue virus (DENV) non-structural protein 1 (NS1). *In vitro:* human dermal endothelial cells exposed to DENV NS1. *In vivo:* Murine model of vascular leak in the dorsal dermis of wild-type C57BL/6 mice	***Permeability:*** Transwell trans-endothelial electrical resistance (TEER) to evaluate the effect of NS1-induced endothelial hyperpermeability in HMEC-1 monolayers. ***Imaging***: Confocal microscopy on HMEC-1 monolayers to assess glycocalyx components (sialic acid, chondroitin sulfate, heparan sulfate, and hyaluronic acid) ***Cytokines***: Human dermal microvascular endothelial cell line HMEC-1 stimulated with DENV2 NS1 and supernatant collected ***Permeability in vivo**:* retro-orbital (RO) injection of IV injection of dextran molecules labeled with a fluorophore (Alexa Fluor 680), which can be quantified via fluorescent scanning	*In vivo*, DENV NS1 but not West Nile virus NS1 triggered localized vascular leak in the dorsal dermis of wild-type C57BL/6 mice. *In vitro*, human dermal endothelial cells exposed to DENV NS1 did not produce inflammatory cytokines (TNF-α, IL-6, IL-8). Blocking these cytokines did not affect DENV NS1-induced endothelial hyperpermeability. DENV NS1-induced endothelial cell-intrinsic vascular leak was independent of inflammatory cytokines, but dependent on endothelial glycocalyx components
***In vitro*** **(Glass)**							
Ebong et al. (26)	*In vitro;* glass Corning slides	Bovine aortic (BAEC) and rat fat pad endothelial cells	Static	Cell media	Cells subjected to conventional or rapid freezing/freeze substitution transmission electron microscopy (RF/FS TEM)	***Imaging (RF/FS TEM*****):** Cells were quick frozen in a Life Cell CF 100 slam freezer by impacting the cell monolayer against a polished sapphire surface cooled to liquid nitrogen temperature. Freeze substitution performed in acetone containing 1% osmium tetroxide. ***Imaging (Immunoconfocal***): Heparan sulfate (HS) and hyaluronic acid (HA) were labeled with anti-HS and HA binding protein, respectively	RF/FS-TEM revealed impressively thick bovine aortic endothelial cell glycocalyx of ~11 μm and rat fat pad endothelial glycocalyx of ~5 μm
***In vitro*** **(Elastomer)**							
McDonald et al. (27)	*In vitro;* 3D cell culture models (silicon elastomer)	Human abdominal aortic endothelial cells	Steady state, uniform, laminar shear stress	Cell media	Cells seeded within 3D straight tube models and subjected to 24 h of 10 dyn/cm^2^ laminar shear stress, investigating the role of glycocalyx in leukocyte adhesion under flow	***Staining***: Primary antibodies (Heparan sulfate, 1:100, Millipore MAB2040 and ICAM-1, 1:200, Santa Cruz sc-8349 monoclonal); Secondary antibody (1:600, Alexa Fluor 488 Anti-Mouse IgG, Molecular Probes, A21206). ***Imaging***: Immunofluorescence staining with laser scanning confocal microscopy	With enzymatic degradation of the glycocalyx, endothelial cells developed a proinflammatory phenotype when exposed to uniform steady shear stress leading to an increase in leukocyte adhesion
Potter et al. (28)	*In vitro/in vivo;* cylindrical microchannels in collagen gel within an elastomer scaffold	HUVECs	Steady state perfusion (gravity-induced pressure head)	Cell media	Murine cremasteric muscle and vasculature exteriorized under anesthesia	***Imaging in vitro**:* micro-particle image velocimetry	Microviscometric analysis of μ-PIV data determines the hydrodynamically relevant glycocalyx thickness, which may be more physiologically relevant and meaningful than the molecular or structural dimension of the glycocalyx measured using direct visualization methods
Yao et al. (29)	*In vitro;* EC monolayers within an acrylic parallel plate apparatus lined with silicon	BAECs, HUVECs	Steady state perfusion (15 dyn/cm^2^)	Cell media	Role of the glycocalyx in both endothelial cell short-term and long-term mechano-transduction responses by using heparinase III to cleave heparan sulfate GAGs on the cell surface	***Staining**:* CellTrace Red-Orange AM (Invitrogen) to detect cellnuclei; VE-cadherin antibody (BD Transduction Laboratories) to detect cell-cell junctions. ***Imaging**:* Cooled charge-coupled device camera (Apogee Instruments) on a Nikon Eclipse TE2000 microscope with a 63 water-immersion objective and MaxIm DLsoftware (Apogee Instruments).	Removing the glycocalyx by using the specific enzyme heparinase III, endothelial cells no longer align under flow after 24 h and they proliferate as if there were no flow present. Glycocalyx is necessary for the endothelial cells to respond to fluid shear, and the glycocalyx itself is modulated by the flow
Bai and Wang (30)	*In vitro;* EC monolayers in a silicon gasket between two transparent polymethylmethacrylate (PMMA) slides	HUVECs	Steady state perfusion (12 dyn/cm^2^ for 24 h)	Cell media	Effects of shear stress on the spatial distribution of the glycocalyx on endothelial cell membranes	***Staining:*** WGA used to bind to N-acetyl-D-glucosamine and sialic acid (SA) component of the glycocalyx. ***Imaging**: Confocal microscopy (Leica Microsystems, Wetzlar, Germany)*.	Following 24 h recovery under shear flow, the glycocalyx reappears predominantly near the edge of endothelial cells. Static and shear flow conditions result in notable changes in the spatial recovery of the glycocalyx, but the difference is not statistically significant

With reported thicknesses of the glycocalyx being in the order of nanometres, imaging modalities form a critical component of any model making an assessment of the glycocalyx *in vivo*, as well as the glycocalyx layer *in vitro*, and this field has evolved considerably. The first imaging of the endothelial glycocalyx by electron microscopy (EM) used the cationic dye ruthenium red (31), with subsequent studies using gold colloids and immunoperoxidase labeling (32). A major disadvantage of these methods was the degradation of the glycocalyx, which occurred with dehydration associated with fixative agents. Many of the methods incorporating EM yielded endothelial glycocalyx thicknesses <100 nm in size (3).

Newer imaging methods to visualize the glycocalyx have since evolved. These include laser scanning confocal microscopy and multi-photon microscopy, with fluorescently labeled antibodies to heparan sulfate or hyaluronic acid binding protein, or wheat germ agglutinin (which binds to sialic acid) to bind to components of the endothelial glycocalyx. In comparison to EM, application of these methods has demonstrated endothelial glycocalyx thicknesses in large blood vessels of 4,300–4,500 nm in the mouse common carotid artery, 2,200 nm in the mouse internal carotid artery, and 2,500 nm in the external carotid artery (33–35). Novel cryogenic protocols combined with electron microscopy have yielded promising results, with one study by Ebong et al. demonstrating that a rapid freezing/freeze substitution method combined with transmission electron microscopy revealed substantial glycocalyx layers on cultured endothelial cells (26). However, more recently, chemical and cryogenic fixation have been shown to lead to substantial collapse of the glycocalyx (36).

Sidestream dark field imaging (SDF) is a non-invasive technique and has been used to analyse the glycocalyx *in vivo*. In a bedside evaluation of the sublingual microvascular glycocalyx in sepsis, acutely ill patients demonstrated damage to the glycocalyx compared to healthy control subjects, with more severe alterations during sepsis (37). One observational study based in Vietnam demonstrated that in patients with Dengue, SDF imaging demonstrated disruption to the glycocalyx, with worse observed glycocalyx injury and higher plasma concentrations of degradation products being associated with worse plasma leakage. The authors reported that in patients with dengue, the perfused boundary region (PBR hf), a surrogate for glycocalyx degradation, was higher in patients with Grade 2 vs. Grade 0 plasma leakage during the critical phase (PBR hf 1.96 vs. 1.36 μm for Grade 2 vs. Grade 0 plasma leakage on days 4–6, respectively, *p* < 0.001) (38). SDF, unfortunately, also has associated drawbacks including that it is prone to impaired visualization of capillaries, as well as interference of moving blood components, such as erythrocytes, with the quality of the image obtained (39).

Importantly, techniques such as confocal microscopy and SDF are applicable both *in vivo* and *in vitro*, whilst the need to fix samples for scanning electron microscopy precludes its use *in vivo*. The pursuit of the optimal imaging modality for studying the glycocalyx remains a field of ongoing research.

## Limitations of Existing *in vitro* Models

**Figure 1:**
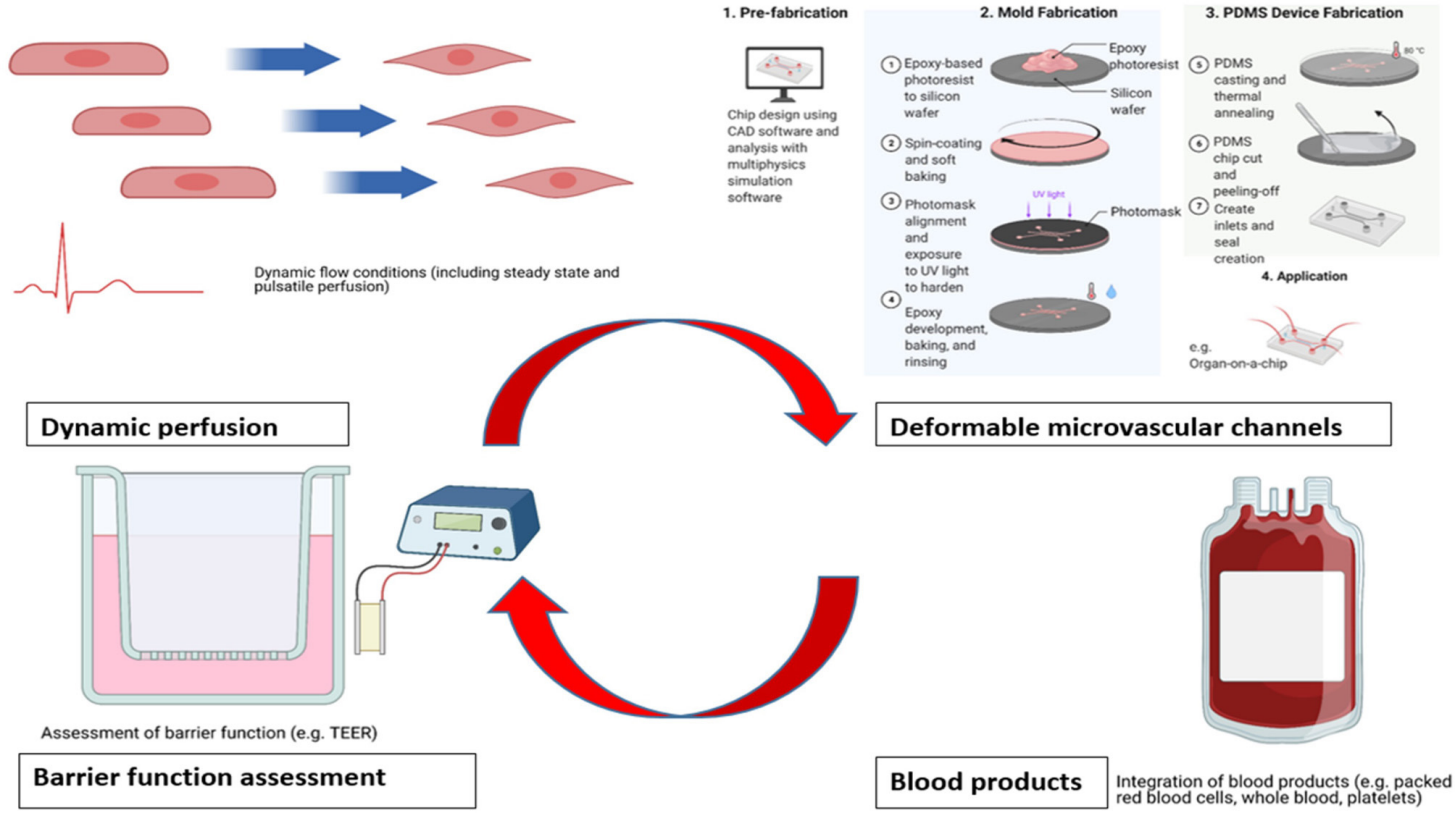
Areas for potential enhancement for existing *in vitro* benchtop models studying glycocalyx pathophysiology. Acknowledgment: *Biorender.com*.

Whilst a number of studies have successfully demonstrated the structure, shedding and function of the endothelial glycocalyx layer across a range of different pathophysiological conditions, there are inherent limitations remaining with current *in vitro* models. These may be approached systematically using categories of (1) flow conditions, (2) materials employed, (3) assessment of endothelial barrier function, and (4) usage of blood products (Figure 1).

### Static vs. Dynamic Flow Conditions

A critical component in the investigation of the glycocalyx layer is the exposure of the cells to dynamic, rather than static, flow conditions that more closely model physiological conditions. The use of static endothelial cell models, usually Transwell systems, readily enables the assessment of endothelial barrier function and its response to chemical or biological stimuli (20). However, the morphology of cultured endothelial cells differs considerably under static compared to perfused conditions, and the thickness of the glycocalyx has been shown to be limited under static conditions, which diminishes its physiological relevance (40). Ueda et al. reported a glycocalyx thickness under static conditions of ~22 nm under static conditions (41), in comparison to Tsvirkun et al. who reported a glycocalyx thickness of ~600 nm when cultured under flow conditions (42). This was further corroborated by Gouverneur et al. who demonstrated that the application of fluid shear stress to cultured HUVECs resulted in a 3-fold higher level of hyaluronan binding protein compared to cells under static conditions. This increased incorporation of hyaluronan may well contribute vasculoprotective properties against inflammation and atherosclerosis to the glycocalyx (4).

Flow-induced shear stress is not only important for the development of the glycocalyx, but it also plays a critical role in maintaining endothelial cell integrity and mechanotransduction (43), particularly in specific cell signaling processes (44). Thi et al. showed that a stable glycocalyx layer is required for the endothelial cell cytoskeleton to respond to shear stress (45), which activates endothelial nitric oxide synthase (eNOS) for the production of nitric oxide (46). This enzyme is critical for modulating the control of vascular tone, leucocyte immigration, and blood clotting (47). Furthermore, the majority of studies conducted under flow conditions have integrated steady-state flow, which provides a constant shear stress on the endothelial cells. However, physiologic pulsatile flow delivers fluctuating shear stress. It has been shown previously that pulsatile flow will vary endothelial cell morphology, enzymatic activity and gene expression, compared to continuous flow (48–51); Uzarski et al. demonstrated that variable shear stress, with temporal changes in pulsatile flow, leads to anti-inflammatory and anti-thrombotic phenotype expression (50), in other words, consistent with the homeostatic vascular endothelium. Furthermore, previous studies have demonstrated that endothelial phenotypes have changed based on pulse frequency (52), pulse amplitude (53), and magnitude of shear stress (54). However, comparative studies of steady-state vs. pulsatile flow specifically on glycocalyx integrity are few. For greater translatability, future studies investigating endothelial and glycocalyx responses to inflammation, coagulation or arterial wall damage should integrate dynamic flow conditions as a priority, such as a comparison between steady state laminar fluid shear stress and pulsatile shear stresses, increased incrementally.

To cite one relevant clinical example, the effect of pulsatile flow on the microcirculation remains a novel field of significant academic inquiry. Koning et al. reported outcomes of pulsatile vs. non-pulsatile flow on microcirculatory perfusion in a clinical study of patients undergoing coronary artery bypass graft surgery. Sublingual mucosal microvascular perfusion was measured using sidestream dark field imaging. The observed reduction in perfused vessel density during aorta cross-clamping was only restored in the pulsatile flow group and increased from 15.5 ± 2.4 to 20.3 ± 3.7 mm/mm2 upon intensive care admission (*P* < 0.01). The median post-operative microvascular flow index was higher in the pulsatile group [2.6 (2.5–2.9)] than in the non-pulsatile group [2.1 (1.7–2.5); *P* = 0.001] (55).

### Material Selection and Channel Dimensions

The materials used in perfused microchannels can have a large influence on cell morphology and the thickness of the glycocalyx developed, as the materials will have different mechanical properties than the native ECM, thereby changing the stiffness and associated mechanical environment stimulation of the endothelial cells. Microfluidics is the technology of fluid manipulation in channels with dimensions of tens of micrometers (56). Selecting a material for a microfluidics application can be a sophisticated process. Flexibility, air permeability, electrical conductivity, non-specific adsorption, cellular compatibility, solvent compatibility and optical transparency are all physical characteristics nominated by Nge et al. as being potentially significant factors affecting choice of material used in microfluidics applications (57). For the purposes of microfluidic models to study the endothelium, biocompatible channel materials that will promote cell adhesion, growth and differentiation with minimal inflammatory response are highly favored (58). Silicon and glass were the original materials used for this purpose. Collagen scaffolds were also used for early *in vitro* models that successfully demonstrated confluent monolayers of endothelial cells. Subsequent studies built on this concept, extending bioremodelable hydrogels (type I collagen) to develop 3D microvascular networks, with user-defined geometries (59). This was followed by a shift to polymer substrates, and in particular, polydimethylsiloxane (PDMS) (57), which is able to be manufactured effectively into small-diameter channels (<1,000 μm) if required and provides sufficient mechanical stiffness to support physiologically relevant flows at this size.

PDMS has also been shown to support more sophisticated, fully endothelialized microvascular networks. Tsvirkun et al. first successfully demonstrated that endothelial cells confined within microchannels and exposed to a physiologically relevant level of fluid shear stress exhibit a glycocalyx that lines the entire lumen of the channels (42). Importantly, PDMS has also demonstrated pressure-induced deformation under flow, which is important for simulating vessel compliance (60). One significant drawback of PDMS is its ability to absorb very small molecules, which may not be desirable for certain applications (e.g., drugs) (61).

Flow channel dimensions, whilst influential on platelet-surface and platelet-platelet interactions, are not widely reported in existing models. The shift from platelet-surface interactions or platelet-platelet interactions occurs as a function of channel size and aspect ratio (height/width) with values of <0.2 are being recommended to achieve constant shear stress, and hence laminar flow (62).

### Assessment of Endothelial Barrier Function

The demonstration of endothelial barrier function is another important consideration and limitation in current models, in order to confirm that the presence of observed structural changes in glycocalyx components are occurring within a functioning endothelium. A critical characteristic of the endothelium is its barrier function; therefore, a model that stipulates that it has replicated the glycocalyx *in vitro* should also demonstrate that its barrier function is maintained. Commonly used examples include direct methods, such as transendothelial electrical resistance (TEER), or indirect methods using a permeability coefficient from transendothelial filtration of tracer molecules (e.g., FITC-Dextran).

TEER measurements have become the gold standard for assessing endothelial monolayer integrity, which typically consist of a volt-ohm resistance meters fitted with “chopstick” or chamber electrodes (63). When serial resistance measurements are taken over time, increasing confluence of the endothelial monolayer is then reflected by stabilization of resistance. This may also be corroborated with an observed increase in confluence, as demonstrated by Ferrell et al. in a microfluidic bioreactor with integrated TEER capability (64). Advantages of this method include its relative simplicity and reproducibility (65); however, it does not demonstrate whether the transition of a molecule of interest across the endothelial barrier, such as a protein, occurs or not (66).

In contrast, assessing endothelial barrier function using permeability studies involve the measured diffusion of a known concentration of analyte from the apical to basolateral side of the membrane over a predetermined period of time, also known as solute flux assays. Commonly used substances to perform this include fluorescein isothiocyanate (FITC)–dextran, which is a 3–5 kDa marker used to measure tight junction permeability (67, 68). A disadvantage of this technique is that it does not accurately represent the microvascular environment, due to static conditions on the apical side of the membrane (63). This is a particularly important drawback to note in the development of models of vasculopathic processes, as it may hinder the capacity of such models to study the impact of pathological stimuli and therapeutic interventions. It should also be noted that maintained barrier function alone is not necessarily an absolute indication of a fully functionally intact glycocalyx. Therefore, in order to address this concern, other surrogates for glycocalyx integrity must be considered. For example, an intact glycocalyx is also a critical regulator of cell membrane expression of gap junction proteins (69). On the other hand, degraded HS from the glycocalyx during injury will cause the dislocation of gap junction alpha-1 protein (Cx43) and malfunction of gap junction channels (69). Assessing immunohistochemical surrogates for glycocalyx integrity, such as Cx-containing gap junction activity by measuring interendothelial spread of gap junction permeable Lucifer Yellow dye (69), is one potential method to circumvent this issue.

### Use of Blood Products

Finally, the glycocalyx modulates adhesion of leukocytes and platelets to the endothelium. Incorporating blood products (such as packed red blood cells, leucocytes, or platelets), allows the study of the interplay between endothelial damage and immuno-inflammatory responses, which is not possible if the perfusate is only cell media. Therefore, an important component in modeling glycocalyx layer structure and function *in vitro* is its exposure to whole blood, blood products, or combinations of the two (70). Whilst there are inherent difficulties in perfusion culture of endothelial cells with whole blood, the consideration of blood-endothelial interactions is nonetheless important. However, the majority of studies investigating the glycocalyx layer *in vitro* have used cell culture media as a perfusate, rather than blood (Table 1). Urner et al. demonstrated that incubation with blood products was associated with increased inflammatory mediator release from endothelial cells (71), whilst McDonald et al. demonstrated that enzymatic degradation of the endothelial glycocalyx in cultured endothelial cells under flow resulted in the development of a proinflammatory phenotype and an increase in leukocyte adhesion (27). Thus, studies using microfluidic devices incorporating RBCs or whole blood are warranted. Anticoagulation is a necessary addition for whole-blood perfusion studies to minimize thrombus formation; however, this limits endothelial-blood studies investigating platelet activation or aggregation (72). Whole blood is also opaque, which makes real-time imaging of blood-endothelial interactions difficult (73); however, diluted blood may still allow for microscopic analysis and therefore retain many advantages of using blood.

The selection of endothelial cells is a significant, influential variable. Dong et al. quantitatively examined different sources of endothelial cells in flow studies. They reported that the number of ultralong Von Willebrand Factor (VWF) strings is endothelial source dependent, with fewer ultralarge VWF strings forming on human coronary artery endothelial cells and human lung microvascular endothelial cells, both used after 4–5 passages, than on primary HUVECs and human umbilical artery endothelial cells (74).

Finally, it has been demonstrated by Lanotte et al. that flow resistance in a microcapillary model could more closely resemble *in vivo* scenarios, with a reduction in the velocity of red blood cells when microcapillaries were coated with a polymer brush to mimic the glycocalyx (75). This has potential implications for red blood cell deformation and mechanotransduction of the endothelial cells and further reinforces the utility of integrating blood products.

## Discussion

There is a significant unmet global need in targeted therapeutics for endothelial dysfunction. To cite one notorious example, Dengue is regarded as the most prevalent and rapidly spreading mosquito-borne viral disease of human beings (76). Whilst the course of this disease is most frequently self-limiting, a small proportion will progress to a syndrome characterized by increased capillary permeability, extravasation of plasma and in some cases, irreversible shock and death. Currently, there are no targeted treatment options available, with supportive care being the cornerstone of management.

Therefore, there is high demand for authentic benchtop models which can examine vascular pathophysiology, as well as opportunities for targeted therapeutic strategies, which includes the glycocalyx. The concept of an “ideal” *in vitro* model is attractive, namely because it carries the benefit of tighter control of experimental variables. In order to advance this field, the four areas identified by the authors to enhance current *in vitro* model design have therefore included the use of dynamic flow conditions, material selection, assessment of barrier function and usage of blood products, as described in section Limitations of Existing *in vitro* Models. Their justification, as well as particular controversies which remain in the study of the glycocalyx *in vitro* vs. *in vivo*, are now discussed as follows.

Firstly, *in vitro* models carry the distinct advantage that the glycocalyx layer and its responses to individual variables may be studied in a highly controlled environment, and therefore they support mechanistic lines of scientific inquiry. In contrast, *in vivo* models offer the distinct advantage of being able to capture critical biological feedback mechanisms, such as those between the glycocalyx and acute inflammatory mediators, which is extremely difficult to replicate *in vitro*. Furthermore, whilst the dimensions of the glycocalyx are highly dynamic and will fluctuate with homeostatic mechanisms, there remains considerable variation in the literature between dimensions described from *in vivo* and *in vitro* models. Variations in measured thicknesses of the glycocalyx are even observed from within the same type of human cell. Chappell et al. reported an average thickness of 878 +/– 612 nm in six *ex vivo* umbilical cords, compared to cultured HUVECs with an average thickness of 29.4 ± 5.8 nm for the dense zone and of 117.9 ± 39.1 nm for the outer zone (40). Previous studies have even declared that the glycocalyx observed *in vivo* did not exist *in vitro* at all, although the conditions under which cells have been cultured should be carefully noted. *One* study by Potter and Damiano described that the glycocalyx observed in microvessels *in vivo* was ~520 nm in thickness, but only ~30 nm in thickness *in vitro* in human umbilical vein endothelial cells under standard cell culture conditions (77). This variation of glycocalyx dimensions in the literature is important, because it effectively forms the “confidence interval” within which the efficacy of targeted endothelial protection strategies on glycocalyx preservation may be determined.

Secondly, another area of controversy is that whilst glycocalyx components may be demonstrated through imaging, this does not necessarily correspond to a functioning endothelium. A critical characteristic of the endothelium is its barrier function; therefore, a model that stipulates that it has replicated the glycocalyx *in vitro* should also demonstrate that its barrier function is maintained. Whilst several methods, including TEER and permeability assays, have been well-documented to demonstrate endothelial barrier function, they are not always included alongside imaging or quantitative studies of the glycocalyx. Their usage should arguably now be the minimum standard in any *in vitro* model, and not an optional inclusion.

Thirdly, and as a corollary from the preceding two points, many models described in the literature have so far concentrated on providing imaging of the glycocalyx, demonstrating alterations in its level of permeability, or studying specific interactions with blood components or proteins of interest. The majority of these have been demonstrated in a static environment. Very few, if any, models have been able to integrate all of these aspects into one model; in other words, demonstrate the glycocalyx visually, along with assessment of its barrier function, in a dynamic flow environment. The ideal model should be able to perform all of these functions simultaneously.

The addition of blood products to *in vitro* microfluidics models represents an attractive frontier to replicate vascular pathophysiological processes, such as thrombosis, more faithfully. However, the study of thrombosis specifically applied to microfluidics devices is notoriously difficult. By definition, microfluidic models are built around the precise control of flow rates and volumes in the order of microlitres, or picolitres. As a result of these minute volumes, it therefore becomes difficult to obtain Reynolds numbers of sufficient magnitude to obtain boundary layer separation and reattachment representative of downstream turbulent flow from a stenotic lesion (78).

The study of coagulation within microfluidics devices is still in its relative infancy. The performance of conventional anticoagulants, such as heparin, which is readily used clinically and *in vivo*, can vary significantly in the setting of microfluidics. Two papers are worthy of note in this regard. Firstly, Harris et al. reported that in a model of human blood being perfused over porcine endothelial cells, there was a strong reduction in platelet surface area coverage and thrombus volume upon treatment of heparinized whole blood with the thrombin inhibitor bivalirudin. This implied that anticoagulation with heparin was insufficient to block thrombin generation in this model (79). Secondly, Ciciliano et al. recalcified citrated blood to allow coagulation to occur, and reported a reduction in channel occlusion with hirudin and heparin in this setup (80).

## Conclusion

The glycocalyx is an essential homeostatic organ and is widely implicated in medical and surgical pathophysiology. It is also an elusive, dynamic structure to investigate. Whilst true dimensions of the glycocalyx may indeed be variable, what remains an essential priority is that *in vitro* model designs advance to the level whereby the endothelium is simultaneously able to be cultured and studied under perfusion, its surface layer is able to be consistently imaged, and its barrier function able to be assessed dynamically. A model of this kind would be of tremendous value in developing targeted therapeutic interventions for vasculopathic conditions.

## Author Contributions

AH, NB, and EW prepared the manuscript. MV, AM, SY, SB, EH, JS, and JF provided expert opinion, critical review, and feedback on the manuscript. All authors contributed to the article and approved the submitted version.

## Conflict of Interest

The authors declare that the research was conducted in the absence of any commercial or financial relationships that could be construed as a potential conflict of interest.
